# Assessment of optometrists^’^ knowledge, skills and practice on cataract: a cross-sectional study from Kisumu County, Western Kenya

**DOI:** 10.1186/s12886-020-01673-w

**Published:** 2020-10-07

**Authors:** Shadrack Muma, Stephen Obonyo

**Affiliations:** 1 Department of Public Health, Maseno University, Po Box Private Bag, Maseno, Kenya; 2 Department of Computing and Informatics, Strathmore University, Po Box Private Bag, Nairobi, Kenya

**Keywords:** Visual acuity, Cataract, Optometrists

## Abstract

**Background:**

The quality of life can be impacted negatively by blindness arising from cataract. The total prevalence of blindness in Kenya is estimated at 0.7%, however cataract contributes almost half (43%) of the total blindness in Kenya. Optometrists are well placed to assess and refer cataract patients. However, little is known on optometrists’ skills, practice and knowledge. Therefore, this study was designed to assess optometrists’ knowledge, skill and practice on cataract in Kisumu, Kenya.

**Methods:**

A cross-sectional study design was used. The study was conducted from June 2019 to August 219 using self-administered questionnaire. Basic socio-demographic characteristics were collected and participants’ knowledge, skills and practice on cataract were investigated. The primary outcome measure was the proportions of participants who identified the questions related to knowledge, skills and practice on cataract. Chi-square analysis was performed to assess the association between demographic characteristics of participants with practice, knowledge and skills.

**Results:**

A total of 49 optometrists with a mean age of 30.4 years and mean duration of practice of 1–10 years were interviewed. Most optometrists had good knowledge on various aspects of cataract. For example (98%) had a good knowledge on the types of cataract. Almost three quarter (75.5%) of the optometrists reported that they could diagnose cataract correctly based on skills. However, half (57.1%) of the optometrists could not identify nuclear cataract. Being a self reported practice and not an observed practice, most optometrists (61.2%) reported that they did not screen patients aged 40 years and above for cataract. Almost half (52.6%) of the optometrists reported that they did a routine eye examination however, they could not justify the significance of examining the crystalline lens for patient above 40 years.

**Conclusion:**

The study established that despite the good level of knowledge among optometrist on cataract, there exist a gap on skills and practice. The results of this study calls for more clinical based activities among optometrists. This will eases diagnosis of cataract and its management with an aim to reduce the burden in Kenya.

## Background

Cataract generally is a group of eye disease characterized by clouding of the eye lens as a result of protein deposits on the natural lens of the eye [1]. Decreased visual acuity is a symptom of cataract, however its absence or presence does not signify cataract. Globally, cataract is the leading cause of blindness and the World Health Organization model on blindness reports that the disease accounts for 6.7 million people being blind [14]. Cataract affect the quality of life for more than 70 million people worldwide [8]. Kenya having a population of 47 million, the prevalence of blindness is estimated at (0.7%) with cataract contributing to (43%) of the total blindness [3]. Although cataract can be treated, early detection is necessary to allow for a follow up until a surgical intervention is carried out.

Cataract impact negatively on the quality of life and may influence daily activities such as driving, walking and economic activities [16]. The burden of cataract can only be reduced through coordination between optometrists in diagnosis and ophthalmologists on surgical interventions [15]. In Kenya, optometrists are the first line eye care providers with a responsibility to conduct a comprehensive eye examination [4]. The national strategic plan for avoidable blindness of 2014–2018 established that optometrists should work closely with ophthalmologists for effective delivery of eye care services [5]. Therefore, optometrists’ roles should not only be inclined towards management of uncorrected refractive error but also in assessment of cataract and other ocular pathologies. Since cataract is a natural occurrence, the prevalence of visual loss may be prevented through early intervention like screening. Therefore, optometrists skills should to be assessed.

A study by Phil et al. [7] in India reported that optometrists screened patients and referred for surgical procedures in hospitals with ophthalmologist. However, almost half (*n* = 1148) (45.8%) of the referred patients had no evidence of cataract, with only 510(20.4%) having cataract after ophthalmologist review. The Kenya Ophthalmic Division defines the roles of optometrists to examine patients, take visual acuity, refract patients and refer patients to ophthalmologists. Similar to United States, where optometrists roles are to examine patients, take visual acuity, refract patients and refer patients to ophthalmologists [11]. In Kisumu, Kenya there is no evidence in the literature about optometrist’s practice, knowledge and skills on cataract. Furthermore, cataract care presents a major challenge when a surgical intervention is not sought at the right time. Therefore this study evaluated optometrist’s knowledge, skills and practice on cataract in Kisumu, Kenya.

## Study rationale

I. With increasing prevalence of cataract in Kenya which can potentially outstrip the current capacity within hospital based cataract care, knowledge of optometrist’s on cataract management is necessary.
II. No study has been done before in Kenya to assess the knowledge, skills and practice of optometrist on cataract.
III. The findings of this study informs on areas that need to be tackled by policy and necessitate for in house courses which will promote knowledge, skills and practice on cataract.

## Methods

### Study area

This study was conducted in Kisumu County, Kenya. Study participants were drawn from seven facilities recognized by the Ophthalmic Division. Kisumu County was chosen as it is the pioneer county to roll Universal Health Coverage [13]. Therefore, the need of optometrists was desirable to facilitate the achievement of Universal Health Coverage objectives. The main economic activity in Kisumu is fishing, a clear indication of prolonged period of exposure to sunlight which is a risk factor of cataract. Kisumu County has a population of 1.2 million [3]. The following facilities were included in the: Sabatia eye hospital, Jaramogi Oginga Odinga Teaching and Referral, Monitor Optical, Optic Ophthalmology Centre, Vision Eye centre, Port Florence Clinic and City Centre Optics.

### Study design

This was a prospective cross-sectional study. The design was more appropriate as it provided accurate account of the characteristics of a particular individual for the purpose of discovering new meaning and describing what exists.

### Study population

Condition for recruitment was based on registration by Optometrists Association of Kenya and working in a facility recognized by the Ophthalmic Division of Kenya. The optometrists included in the study were aged between 25 and 36 years. The sample was derived from a target population of 149 registered optometrists with the Optometrists Association of Kenya. The participants were recruited from June 2019 to August 2019. Using a standard normal deviate of 1.96; a conservative proportion of 0.5 in the target population (given no previous knowledge, skills and practice reference in Kenya) the level of accuracy required was fixed at 0.05; a sample size of 49 participants (using a base population of 149) was determined using formula: $n=\frac{N{Z}^2P\Big(1-P}{d^2\left(N-1\right)+{Z}^2P\left(1-P\right)}$ Unregistered optometrists were excluded from the study.

### Participant recruitment

Participant’s recruitment was done through an invitation letter sent to the optometrists explaining what the study was all about and the significance. Three reminders were sent to the participants through their email informing them on when the study will be conducted.

### Sampling procedure

Simple random sampling was conducted to recruit participants from the respective clinics (*n* = 7) and demographics. From a sampling frame of *n* = 149 optometrists, a sample size of 49 was derived. The researcher listed the target population and assigned consecutive numbers from 1to 149. An online random number calculator was used to generate numbers to be included in the study. A total of 49 random numbers were selected from a sampling frame which constituted the sample size. The research instrument was pretested among 5 optometrists. A pilot study with a sample of a tenth of the total sample is appropriate for a pilot study [6]. Therefore a tenth of the total sample was 5. The participants included in the pilot study were not included in the actual study since they could influence the outcome the study results. The researcher tested reliability using Cronbach’s alpha (0.874, 0.929 and 0.926 for knowledge, skills and practice questionnaire respectively) and validity using a Pearson correlation coefficient (0.000 < 0.05, *N* = 5).

After potential participants were identified, they consented and a copy of the signed consent was given to them. The response rate was 100% and this was enhanced by maintaining a constant contact between participants and the researcher at all time during the study period.

### Data collection instrument

The aim of this study was to assess the optometrists knowledge, skills and practice on cataract as it is one of the major cause of blindness. Self administered questionnaire was used as it had the potential to reach out to a large number of respondents within a short time. Secondly the questionnaire gave the respondents adequate time to respond to the items. Seven research assistants were recruited to administer the questionnaires to the respondents. The research assistants were trained on research ethics and how the process was to run. The questionnaires were given to only optometrists practicing in the seven recognized Ophthalmic Division facilities in Kisumu County, Kenya.

The questionnaires consisted of closed ended questions aiming to explore the participant’s knowledge, skills and practice on cataract. Information collected included: socio demographic characteristics, knowledge of various aspects of cataract, skills and practice of optometrists on cataract. The study had 16 structured questions broadly around respondent’s demographic characteristics (3 questions), knowledge on cataract (4 questions), skills on cataract (7 questions) and practice on cataract (2 questions). The questions had two to three responses of aware, neutral and not aware on a three point Likert scale. One open ended question explored the reason for referral of cataract patients by optometrists. The questionnaires took 20 min to complete. A composite scale ranging from 20 to 100 was adopted with knowledge, skills and practice level categorized as: low (score of 20–40), medium (score of 41–79) and high/good (score of 80–100). The participants were to identify the type of cataract based on the seven photographs shown to them. The questionnaires were administered when participants were free and collected back each and every evening. The questionnaires were given to the participants with an option to seek clarification in case of any difficulty from the lead researcher.

### Analysis

Frequency distributions of all socio demographic characteristics and the proportion of participants who identified all items related to skills, knowledge and practice were calculated. A chi-square was used to calculate the association between demographic characteristic and knowledge, skills and practice. We conducted multiple logistic regression analysis to compare the knowledge, skills and practice with demographic characteristics. We calculated odds ratios (ORs) and 95% confidence intervals (CIs). Thematic analysis was conducted to analyze the question on reason for referral. There was no missing data as all data obtained were stored manually and in the computer. Statistical Package for Social Sciences version 17 software was used to analyze the data. Values of *p* < 0.05 were considered statistically significant.

## Results

### Demographic characteristics

Male constituted (63.3%) of the participants interviewed with female being (36.7%). The mean age of the participants was 30.4 (SD 6.9) with an age range of 25–36 years. The maximum year of practice for the participants was 10 years with a mean of 5.4 (SD 4.2).

### Knowledge on cataract

Most optometrists could correctly answer questions related to knowledge on cataract. For example (61.2%) could correctly define cataract; almost half (47.0%) had knowledge on complication of cataract; symptoms of cataract (93.6%) and types of cataract (98.0%). However, only (12.2%) reported that inability is a complication of cataract; floaters as a symptom of cataract (4.1%) and traumatic cataract (20.1%) (Table 1).

**Table 1:** Knowledge of optometrists on cataract (*N* = 49)

Variable	Number (%)
**Cataract definition**	
Opacification of the crystalline lens of the eye	30 (61.2)
Presence of a white coating on the lens	12((24.5)
Poor vision in the eye	6 (12.2)
Absence of the lens of the eye	1 (2.0)
**What types of cataract do you know?**	
Sub-capsular cataract	20 (40.8)
Cortical cataract	30 (61.2)
Traumatic cataract	10 (20.4)
Nuclear Cataract	48 (98.0)
**Complications of cataract**	
Blindness	47 (95.9)
Blurred vision	13 (26.5)
Inability to read	6 (12.2)

Table showing the number of respondents who could correctly define cataract, knew the types of cataract and were aware of the complication of cataract

### Skills of optometrists on cataract

Majority of the respondents (75.5%) used their skills to diagnose cataract based on reduced visual acuity and funduscopy examination results. One hundred percent of the respondents agreed that funduscopy was significant in diagnosis of cataract. Fewer respondents (4.1%) reported that they use pen torch for assessment of cataract. On the assessment of crystalline lens, majority of the respondents (83.7%) stated that they always do direct ophthalmoscopy with an half (59.2%) doing indirect ophthalmoscopy (Table 2). Majority of the respondents could correctly identify the types of cataract from the stereoscopic photos. For example (65.3%) could correctly identify sub-capsular cataract, (77.5%) cortical cataract and (70.8%) for traumatic cataract. However nuclear cataract was only identified by (42.90%).

**Table 2:** Skills of optometrists on cataract

Variable	Number (%)
**How do you make a Diagnosis of cataract?**	
Slit lamp assessment	10 (20.4)
Pen torch assessment	2 (4.1)
Reduced vision and Funduscopy	37 (75.5)
**Do you think Funduscopy is important?**	
Yes	49 (100)
To make a diagnosis of cataract	49 (100)
For Follow up	30 (61.2)
To determine which surgery	20 (40.8)
**How can lens be assessed**	
Direct ophthalmoscope	41 (83.7)
Indirect ophthalmoscope with 90D and 78D.	29 (59.2)

Table above shows the skills of the respondents on diagnosis, significance of Funduscopy and lens assessment

### Practice of optometrists on cataract

**Fig. 1:**
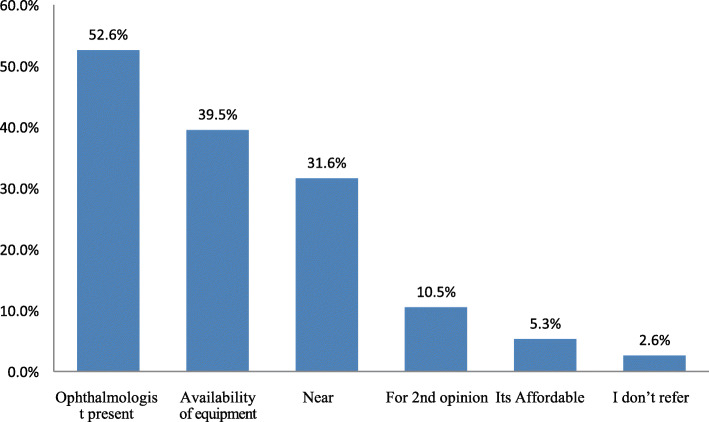
Reasons for Referral

Majority of the respondents (61.2%) stated that they do not examine all patients over 40 years for cataract. However, the 38.8% of the respondents who could screen, only (47.4%) knew the importance of screening while (52.6%) stated that they just do screening as a routine eye examination. Half (52.6%) of the respondents reported that they always refer patients to a particular hospital due to availability of ophthalmologist and availability of ophthalmology equipment (39.5%). However, (2.6%) did not refer patients (Fig. 1).

### Association between demographic characteristics and knowledge on cataract

There was good knowledge among participants aged 25–30 years (77.4%) as compared to those aged 31–36 years (22.6%). Based on the duration of practice, participants who had practiced for less than 5 years had a good knowledge of cataract (76.0%) compared to those who had practiced for more than 5 years (12.0%). The composite awareness score showed that most respondents had a good knowledge on cataract (78.9%). There was no statistically significant difference on knowledge of cataract based on qualification (71.4%) for bachelor compared to (28.6%) diploma. However, age group and duration of practice were significantly different based on knowledge of cataract (*p* < 0.001, *p* = 0.033 respectively) (Table 3).

**Table 3:** Associations between demographic characteristics and knowledge on cataract

Variable	Knowledge		OR(95% C.I)	***P***-value
	Good	Poor		
**Age group (*N*** **= 49)**				
25–30	24 (77.4%)	4 (22.2%)	12.0 (3.0–51.6)	< 0.001
31–36	7 (22.6%)	14 (77.8%)	1.0	
**Duration of Practice**				
< 5	19 (76.0%)	6 (25.0%)	1.0	
6–9	3 (12.0%)	5 (20.8%)	0.2 (0.0–0.9)	0.033
> 10	3 (12.0%)	13 (54.2%)	0.1 (0.0–0.3)	< 0.001
**Qualification**				
BSc Optometry	25 (71.4%)	7 (50%)	2.5 (0.7–9.2)	0.155
Dip Optometry	10 (28.6%)	7 (50%)	1.0	

Table above shows the association between demographic characteristics and the respondent’s knowledge on cataract with a confidence interval and a p value

### Associations between skills on cataract and demographic characteristics

Concurrently, on skills a relatively similar scenario was observed. Based on duration of practice, half of the participants who had practiced for less than 5 years (57.1%) had good skills on cataract compared to those who had practiced for more than 10 years (21.4%). The average composite awareness score was (40%), a clear indication of deficit on skills among respondents on cataract. However, there was no statistically significant difference on skills of cataract for those who had practiced for 6 to 9 years (*21.4%*) compared to those who had practiced for less than 5 years (51.7%) (Table 4).

**Table 4:** Associations between demographic characteristics and skills on cataract

Variable	Skills		OR(95% C.I)	***P***-value
	Good	Poor		
**Duration of practice**				
< 5	16 (57.1%)	6 (28.6%)		
6–9	6 (21.4%)	4 (19.0%)	0.4 (0.1–1.8)	0.256
> 10	6 (21.4%)	11 (52.4%)	0.2 (0.1–0.8)	0.019
**Qualifications**				
BSc Optometry	27 (77.1%)	9 (64.3%)	1.9 (0.5–7.2)	0.357
Dip Optometry	8 (22.9%)	5 (35.7%)	1.0	

Table above show the association between demographic characteristic and the respondent’s skills on cataract with a confidence interval and the p value

### Association between demographic characteristics and practice

On practice, proportion of participants who had practiced for less than 5 years and between 6 to 9 years was the same (35.7%). However, there was no statistically significant difference on the qualification of the participants and those who had practiced for more than 10 years (*p* = 0.202, *p* = 0.494) respectively (Table 5).

**Table 5:** Associations between practice and demographic characteristics

Variable	Practice		OR (95% C.I)	***P***-value
	Good	Poor		
**Duration of practice**				
< 5	10 (35.7%)	12 (57.1%)	1.0	
6–9	10 (35.7%)	3 (14.3%)	4.0 (0.9–18.6)	0.069
> 10	8 (28.6%)	6 (28.6%)	1.6 (0.4–6.2)	0.494
**Qualifications**				
BSc Optometry	18 (64.3%)	17 (81.0%)	0.4 (0.1–1.6)	0.202
Dip Optometry	10 (35.7%)	4 (19.0%)	1.0	

Table above shows respondents association between demographic characteristics and practice with a confidence interval and a *p* value

## Discussions

Cataract in Kenya, just like in other countries in sub-Saharan Africa remains a devastating condition. It is the leading cause of avoidable blindness worldwide, with nearly 50–90% true cataract patients remaining undiagnosed [12]. Kenya being a developing country, the health system has not reached the threshold hence influencing the attention towards eye care. There was good knowledge of optometrists on various aspects of cataract like definition and types with a gap on skills and practice. Practice and skills on cataract is still a challenge among optometrists in Kisumu County. Therefore a lot of attention should be directed towards improving optometrists’ skills and practice on cataract.

The study established that most optometrists could correctly define cataract, identify various types of cataract, symptoms of cataract and complications of cataract. This is a clear implication that optometry training in Kenya covers both optics and pathology of the eye. In this study, (95.9%) of optometrists were aware of the ocular complications arising from cataract. There was a significant relationship between knowledge on cataract, age and duration of practice. Optometrists that had practiced for less than 5 years were associated with good knowledge on cataract. This high level of awareness on conditions associated with cataract is important as it motivate optometrist to diagnose and refer patients to ophthalmologists. Better knowledge among young optometrist could be explained by the fact that they recently graduated from optometry training institution. Therefore the old graduates might have lost a lot of theoretical facts since graduation hence finding it difficult to articulate facts. There was no statistically significant association between the qualification with good knowledge, skills and practice on cataract. This could be explained by the fact that the only difference in training between bachelor and diploma in optometry is duration of training. This study established that despite the good level of knowledge among optometrist, there exists a gap in skills in interpretation of type of cataract.

On assessment of skills and practice among optometrists on diagnosis and treatment of cataract, the study found that (75.5%) of the participants were diagnosed of cataract based on reduced visual acuity. This is comparable to what was found in a study at Light house eye hospital in which (90.6%) of all cataract diagnosis was based on reduced visual acuity without consideration of other factors [9]. Visual acuity may not be sufficient to ascertain cataract as there are several ocular related conditions associated with reduced visual acuity. However, on clinical practice combining funduscopy and reduced visual acuity will ascertain the presence or absence of cataract. Hence the optometrist’s scope of practice in Kenya should not only be inclined towards uncorrected refractive error, but also on pathological related conditions. The study established that nearly half of the optometrists referred cataract patients mainly for ophthalmologic review. The choice of hospital where patients were referred to depended on availability of ophthalmologist and cataract equipment. A study in South Africa reported that cataract management has been prioritized and optometrists are enlightened on diagnosis and referral [10]. However, a lot of attention should be directed towards skills and practice of optometrists on cataract management in developing countries.

In this study (61.2%) of the optometrists reported that they did not screen patients age 40 years and above who attended eye clinics. This was possible because optometrists find it hard to relate with other cadres and as a results they do not see the need of referring a patients to the ophthalmologists. However, we also established that (52.6%) of the optometrists who screened patients were not aware of what they were looking for. A similar scenario was reported among optometrists (50.0%) who referred patients above 40 years but were not aware of the reasons for referral [2]. The absence of screening awareness is probably attributed to lack of interest in pathology of the eye with bias towards optics. The thematic analysis showed that each and every optometrist had a reason for referral and the reason for the low referral could be linked to fear of being criticized by the ophthalmologist. In the Kenyan context, optometrists’ roles are confusing as they are capable of performing most procedures in relation to their scope of training. However, the Ophthalmic Division has stipulated the roles of optometrists in eye care delivery but other eye care cadres still assert that optometry is pure optics. So this translate that the burden of cataract may rise in Kenya as the first line eye care providers who meet majority of the patients are inclined towards optics. Hence, the Ophthalmic Division should streamline funds in management of eye care services and ensure that they strengthen the roles of optometrists. The funds will be useful in enabling optometrists to do screening for patients and refer for surgical intervention in case of cataract. The possible reason for the low skills among optometrists is attributed to reduced interest by the practitioners due to the division frustrations.

This study had certain limitations: first, there are more than 300 optometrists in Kenya although only 149 are registered with Optometrists Association of Kenya. The sample size would have been larger if the author would have included all optometrists, however being that they are not interested in joining the association for reasons best known to them, accessing their email addresses proved futile. At the same time, the Ophthalmic Division does not allow unregistered optometrists to engage in any clinical activity. Secondly, as the respondents were only optometrists, the view of the Ophthalmic Division was not included in the study. Getting the opinion of stakeholders would have improved the study. As the division includes most stakeholders, they could have been asked on what is there thought on inclusion of optometrists in the division, roles of optometrists on cataract management and if they think incorporating optometrists in management of cataract will influence the prevalence of cataract. Future studies to be conducted should include the Ophthalmic Division, ophthalmologists in Kenya to confirm the study findings. Third, using questionnaires could have lead to bias as the optometrists could have over reported while other participants could have underreported.

## Conclusions

The present study demonstrates low skills and practice on cataract among optometrists in Kisumu County, Kenya. Therefore, continuous training of optometrist on cataract is necessary. This can be done through supportive supervision, continuous medical education at health facilities and regular skills update workshops. Encourage optometrists to routinely check the lens of all patients above 40 years attending eye clinics. Encourage optometrist to counsel cataract patients as this improves compliance. There is need for ophthalmologists to inform optometrists on the visual acuity score to refer a cataract patient for a surgical intervention. This will ensure that the burden of cataract is greatly reduced due to improved skill development and knowledge among optometrists. Optometrists practice on management strategies like direct ophthalmoscopy, indirect ophthalmoscopy and funduscopy should be strengthened to ensure accurate diagnosis of cataract.

## Supplementary information

**Additional file 1.**

## Data Availability

The dataset for the optometrists generated and analyzed during the current study are available from the corresponding author upon reasonable request.
