# Supplementary material for: Assessment of optometrists’ knowledge, skills and practice on cataract: a cross-sectional study from Kisumu County, Western Kenya
Source: BMC Ophthalmol. 2020 Oct 7;20:401. doi: 10.1186/s12886-020-01673-w (PMC7542908; doi:10.1186/s12886-020-01673-w)
Supplement: Supplementary file 1 — Additional file 1. [file 12886_2020_1673_MOESM1_ESM.docx]

1. **Social-Demographic characteristics (N=49)**

- Age
- Gender
- Years of practice

**Knowledge on definition, signs, symptoms and complication of cataract**

1. **Cataract definition**

- Opacification of the crystalline lens of the eye
- Presence of a white coating on the lens
- Poor vision in the eye
- Absence of the lens of the eye

1. **What types of cataract do you know?**

- Nuclear cataract
- Sub-capsular cataract
- Cortical cataract
- Traumatic cataract

1. **Symptoms of cataracts**

- Reduced Visual acuity
- Light sensitivity
- Blurred vision
- Red eye Floaters
- Others*

1. **Complications of cataract**

- Blindness
- Blurred vision
- Inability to read

**Knowledge on diagnosis of cataract**

1. **How do you make a Diagnosis of cataract?**

- Reduced vision and Funduscopy
- Slit lamp assessment
- Pen torch assessment

1. **Do you think Funduscopy is important?**

- No
- Yes

1. **Why?**

- To make a diagnosis of cataract
- To determine which surgery
- For Follow up

1. **How can lens be assessed?**

- Direct ophthalmoscope
- Indirect ophthalmoscope with 90D and 78D

**Screening patients for Cataract**

| 1. **Do you examine all patients over 40 years for cataract?(N=49)** |
| --- |
| - Yes |
| - No |
| 1. **Why do you screen patients over 40 years (N=19)** |
| - Those who knew importance of screening. |
| - Those who screened as routine eye examination.   **Reasons for screening**   1. **Reasons for screening patients for cataracts**  - Age is related to development of cataract Asymptomatic - Race common in blacks  1. **Why don’t you screen patients for cataract**  - Long queues of patients Lack of adequate personnel - Examination according to patient complaints - Lack of time |
